# Early Changes in CT Perfusion Parameters: Primary Renal Carcinoma Versus Metastases After Treatment with Targeted Therapy

**DOI:** 10.3390/cancers11050608

**Published:** 2019-04-30

**Authors:** Alice C. Fan, Vandana Sundaram, Aya Kino, Heiko Schmiedeskamp, Thomas J. Metzner, Aya Kamaya

**Affiliations:** 1 Division of Oncology, Department of Medicine, Stanford University School of Medicine, Stanford, CA 94305, USA; 2 Stanford Cancer Institute, Stanford University School of Medicine, Stanford, CA 94305, USA; 3 Quantitative Sciences Unit, Department of Medicine, Stanford University School of Medicine, Stanford, CA 94304, USA; 4 Department of Radiology, Stanford University School of Medicine, Stanford, CA 94305, USA; 5 Siemens Medical Solutions USA, Inc., Malvern, PA 19355, USA; 6 Department of Urology, Stanford University School of Medicine, Stanford, CA 94305, USA

**Keywords:** CT perfusion, renal cell carcinoma, metastases, targeted therapy, biomarker, therapeutic response

## Abstract

Computed tomography (CT) perfusion is a novel imaging method to determine tumor perfusion using a low-dose CT technique to measure iodine concentration at multiple time points. We determined if early changes in perfusion differ between primary renal tumors and metastatic tumor sites in patients with renal cell carcinoma (RCC) receiving targeted anti-angiogenic therapy. A total of 10 patients with advanced RCC underwent a CT perfusion scan at treatment baseline and at one week after initiating treatment. Perfusion measurements included blood volume (BV), blood flow (BF), and flow extraction product (FEP) in a total of 13 lesions (six primary RCC tumors, seven RCC metastases). Changes between baseline and week 1 were compared between tumor locations: primary kidney tumors vs metastases. Metastatic lesions had a greater decrease in BF (average BF difference ± standard deviation (SD): −75.0 mL/100 mL/min ± 81) compared to primary kidney masses (−25.5 mL/100 mL/min ± 35). Metastatic tumors had a wider variation of change in BF, BV and FEP measures compared to primary renal tumors. Tumor diameters showed little change after one week, but early perfusion changes are evident, especially in metastatic lesions compared to primary lesions. Future studies are needed to determine if these changes can predict which patients are benefiting from targeted therapy.

## 1. Introduction

Patients with metastatic renal cell carcinoma (RCC) may be treated with 13 potential FDA-approved targeted therapies, of which, six are multi-tyrosine kinase inhibitors (TKIs) directed against the vascular endothelial growth factor receptor 2 (VEGFR2) [1,2,3,4,5,6,7,8,9,10,11,12]. Once a treatment is selected, the treatment response is typically first assessed three months after initiating treatment using computed tomography (CT) Response Evaluation Criteria in Solid Tumors (RECIST) 1.1 measurements [13]. During this time, 10–20% of patients with metastatic RCC will continue to progress while suffering adverse effects that are common with treatment (e.g., fatigue, diarrhea, nausea, hemorrhage, liver toxicity) [14]. An alternative imaging method at an earlier time point to determine response to therapy is highly desirable to identify those who are not responding in order to allow them the opportunity to switch to a potentially more effective therapy sooner.

Using CT perfusion, one can visualize and quantify blood perfusion in tissue [15,16]. Low-dose contrast-enhanced CT images are obtained over multiple (25–30) time points during which an iodine-based contrast agent passes through the vasculature and tissue, allowing quantitative analysis of blood flow, blood volume, and mean transit time. CT perfusion is well established in stroke imaging but is a relatively new method of imaging abdominal tumors such as RCC. Due to its highly vascularized nature, renal cell carcinoma is especially amenable to visualization with CT perfusion [17,18,19,20,21,22]. CT perfusion is not currently widely used in RCC because the standard accepted method of evaluating tumor response is to use size measurement and RECIST criteria. Since most RCC therapies are directed against angiogenesis, we hypothesize that tumor vascularity could decrease with successful treatment. Moreover, in RCC, targeted therapy can have a greater effect on metastatic sites than the renal primary tumor [23]. Therefore, we compared early changes in vascularity in the primary tumor and metastatic sites. We selected an early 1-week time point to measure perfusion changes because targeted treatment reduces tumor vasculature, decreases proliferation, increases apoptosis and immune cell infiltration as early as 1–7 days after initiating treatment in preclinical and translational studies [24,25,26,27,28,29]. We sought to describe early changes in perfusion and determine if there were any differences in perfusion between primary renal cell carcinoma tumors compared to metastatic lesions from RCC.

## 2. Results

Our analysis included 10 patients with advanced RCC who required treatment with anti-angiogenesis agents. Five patients had primary renal masses, one patient had both a primary renal mass and a metastatic lesion, and four patients had metastatic RCC lesions (in single or multiple sites, including adrenal, pancreas, lung, liver, and soft tissue). Patient demographics of tumor type and location, and administered treatment are shown in Table 1. A total of 13 lesions were analyzed: Six with primary renal masses and seven with metastases.

**Figure 1:**
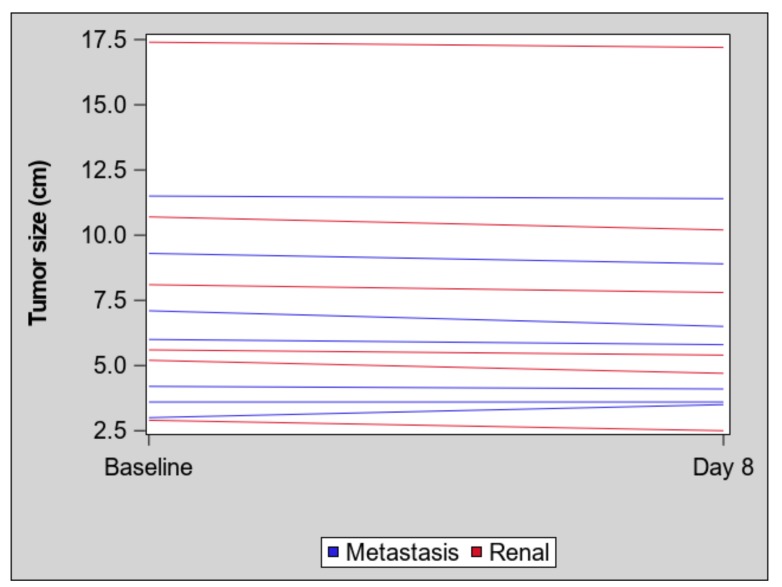
No significant change in tumor size by tumor location after eight days of treatment. Spaghetti plot of the maximum diameter (cm) of each tumor (primary renal mass: red; metastases: blue) at baseline and eight days after initiating treatment. Each line represents a single mass.

At 12 weeks, four (67%) of the renal masses and two (29%) of the metastasized masses had stable RECIST measurements; two (33%) of the renal masses and five (71%) of the metastasized masses had RECIST measurements consistent with progressive disease. There was no statistically significant difference by tumor location for the difference in tumor size between the baseline and the eight-day scan (*t*-test *p*-value = 0.17), see Figure 1.

**Figure 2:**
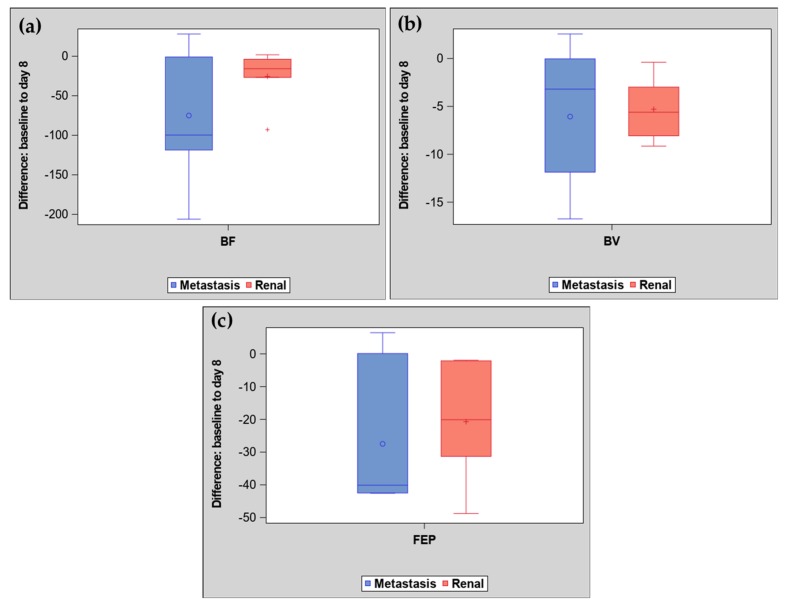
Distribution of differences in computed tomography (CT) perfusion parameters by tumor location. (**a**) Changes between baseline and day eight in blood flow (BF). (**b**) Changes between baseline and day eight in blood volume (BV). (**c**) Changes between baseline and day eight in flow extraction product (FEP). BF, BV and FEP were measured with CT perfusion in patients with primary and metastatic renal cell carcinoma treated with an anti-angiogenic therapy.

**Figure 3:**
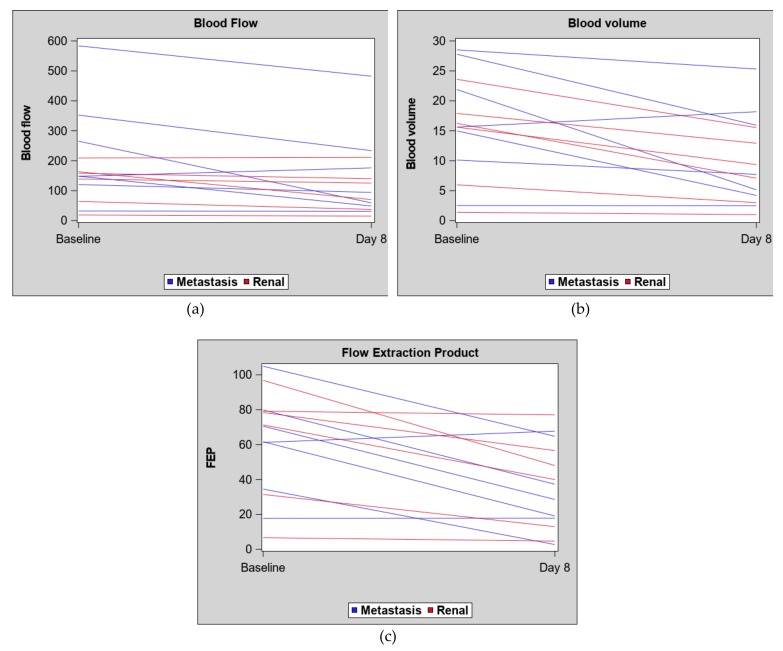
Spaghetti plots for each CT perfusion measure by tumor location: Metastasis versus primary renal mass. (**a**) blood flow (mL/100 mL/min); (**b**) blood volume (mL/100 mL); (**c**) flow extraction product (mL/100 mL/min).

There was considerable variation in blood flow (BF), blood volume (BV) and flow extraction product (FEP) at each time point and in differences in BF, BV and FEP from baseline to the eight-day scan for both kidney and metastatic masses, see Figure 2 and Table 2. While metastatic masses showed a greater decrease in the average BF (BF difference (mean + SD): −75.0 ± 81) than kidney masses (−25.5 ± 35), there was no statistically significant difference in BF difference by tumor location (Wilcoxon rank sum test *p* = 0.28), see Figure 2 and Figure 3.

**Figure 4:**
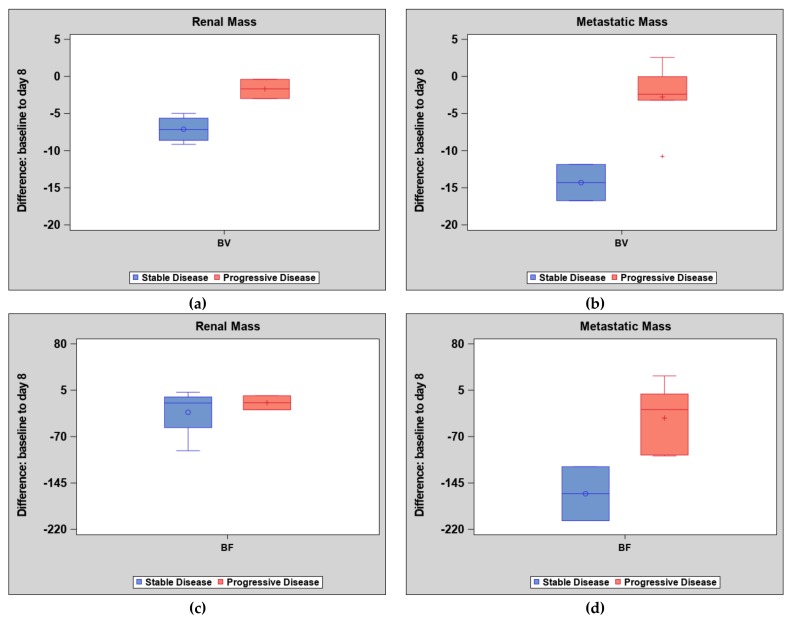
Blood volume and blood flow changes by clinical response and tumor location. (**a**) Differences in blood volume (BV) in primary renal lesions on day eight of treatment with tyrosine kinase inhibitor in patients who had either stable or progressive disease by RECIST 1.1 criteria at 12 weeks post-treatment. (**b**) Differences in blood volume (BV) in metastatic renal cell carcinoma lesions. (**c**) Differences in blood flow (BF) in primary renal lesions on day eight of treatment. (**d**) Differences in blood flow (BF) in metastatic lesions. Note that stable disease shows a larger decrease in BV in both primary kidney masses and metastatic disease compared to progressive disease and a greater decrease in BF is seen in stable metastatic disease compared to progressive disease.

**Figure 5:**
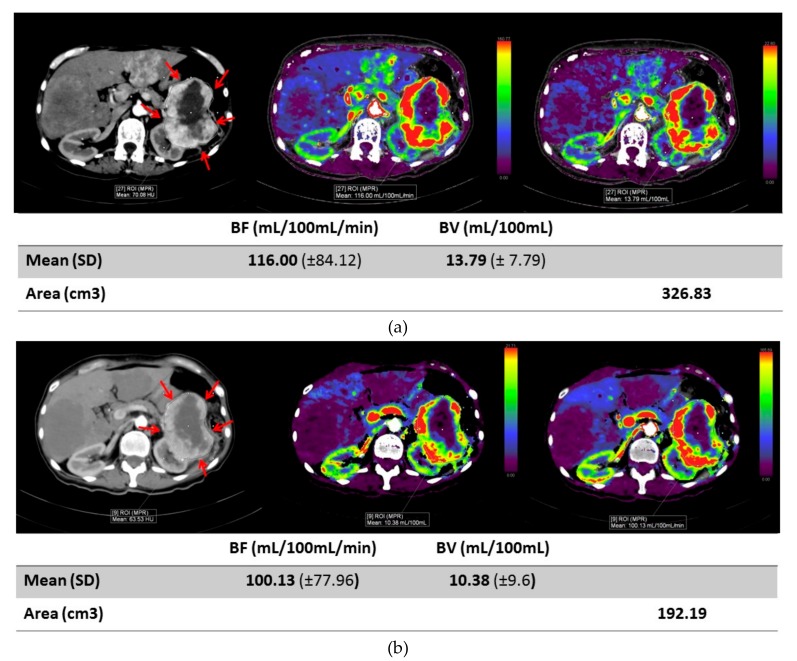
Images of a primary renal lesion. (**a**) Axial contrast enhanced image and CT perfusion blood flow image before therapy of left renal mass (red arrows). Blood flow was measured to be 116 mL/100 mL/min and blood volume was 13.79 mL/100 mL. (**b**) An axial contrast enhanced image and CT perfusion image of same renal mass eight days after therapy shows a mild decrease in blood flow to 100.13 mL/100 mL/min and blood volume to 10.38 mL/100 mL, but with stable size of mass. Standard deviation (SD) for each measurement is shown on the table below the figure.

**Figure 6:**
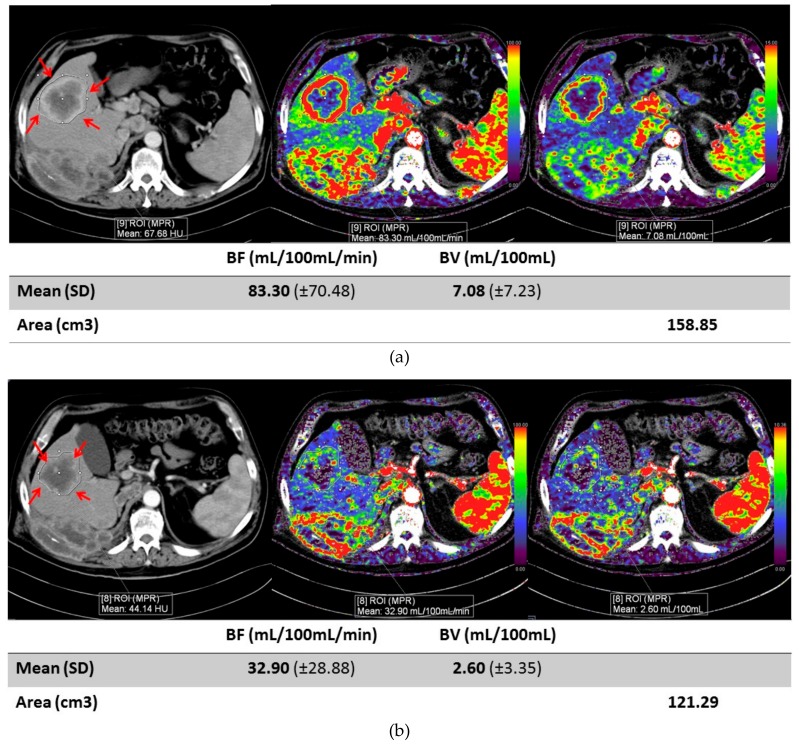
Images of a metastatic lesion. (**a**) Axial contrast enhanced image and CT perfusion images before therapy in patient with renal cell carcinoma metastases to the liver (red arrows). Blood flow is 83.3 mL/100 mL/min and blood volume is 7.08 mL/100 mL. (**b**) Axial contrast enhanced image and CT perfusion image of same patient eight days after therapy shows dramatic decrease in blood flow to 32.90 mL/100 mL/min and blood volume decrease to 3.35 mL/100 mL. Standard deviation (SD) for each measurement is shown on the table below the figure.

There was no statistically significant association between tumor location and clinical response (stable or progressive disease) (OR: 4.4 (90% CI: 0.43–60.54), *p* = 0.42). Patients with stable disease had a greater decrease in BV difference for both kidney and metastatic masses compared to patients with progressive disease. Although not statistically significant, the magnitude of change was greater in metastatic masses compared to primary kidney masses, see Figure 4. Representative images of a primary renal lesion and metastatic lesions are shown, see Figure 5 and Figure 6.

## 3. Discussion

Patients with advanced solid tumors rely upon tumor diameter measurements to determine response to treatment. Patients with RCC would benefit from imaging biomarkers that can determine the response to targeted therapy at an earlier time-point than 12 weeks; we selected patients treated with anti-angiogenesis agents as a tractable patient population for this initial study. We observed that early changes in BF and BV in advanced RCC patients were of greater magnitude in patients with stable disease compared to progressive disease. While we were unable to demonstrate statistical significance, our result is consistent with preclinical data in which successful anti-angiogenic treatment has been shown to inhibit VEGFR2, decrease tumor vessel number and density based on immunohistochemistry in xenograft and preclinical models [30,31,32,33]. In clinical studies of RCC, CT perfusion has been performed at five- and 10-week time points after initiating treatment, but we are the first to demonstrate that changes can be detected as early as eight days after treatment; however, whether early changes can reliably predict clinical outcome will need to be validated in a larger study. Nonetheless, we have demonstrated that measuring early changes in CT perfusion is possible.

For patients with advanced RCC, the goal of systemic treatment is to prevent overall tumor growth at all involved sites to reduce overall morbidity and mortality. In metastatic sites, tumor stabilization or shrinkage is required to reduce or prevent symptoms and preserve vital organ function. For the primary kidney lesions, there is also an additional goal to control the lesion to prevent specific morbidity from pain or acute bleeding [34,35]. Therefore, we specifically evaluated the ability of CT perfusion acquisitions to measure changes in kidney lesions compared with distant metastases. Although we did not reach statistical significance, we found a larger decrease in BV in metastatic tumor sites compared to primary renal tumors in patients with stable disease. Our work suggests that early perfusion changes, especially in metastatic lesions, may help determine if patients are benefiting from targeted therapy. To our knowledge, no other study to date has examined the difference in response to treatment of primary RCC compared to metastatic disease using CT perfusion.

There were several limitations to our study. First, we included patients with advanced cancer (receiving up to four previous lines of therapy). Therefore, the best response in this population was “stable disease”, as no patient achieved significant tumor shrinkage at 12 weeks (i.e., we did not have any patients classified as a partial or complete response). We required that patients receive more than half the expected doses of chemotherapy; therefore, lack of tumor shrinkage was not due to compliance with taking medication but more likely due to the biology of the tumors. It is possible that patients who achieve a partial or complete response might have even greater decreases in vascularity measurements; however, we were not able to show this since we did not have patients who met this criterium. Lastly, this pilot study was a small cohort, intended to explore the possibility of observing changes in CT perfusion parameters. Larger studies in select patient populations will be needed to assess if vascularity measurements can be used for early therapeutic monitoring.

## 4. Materials and Methods

### 4.1. Study Protocol

The study was approved by the institutional review board at Stanford School of Medicine and was conducted in accordance with the principles of the Declaration of Helsinki and the International Conference on Harmonisation guidelines for Good Clinical Practice. The study is listed on clinicaltrials.gov (identifier NCT01926990). All the patients provided written informed consent before study entry. In this prospective pilot study, patients were eligible if they had advanced RCC (stage 3 or 4), were scheduled to receive systemic treatment, had adequate renal function, and had no conflicting allergy. Generally, it is a standard of care to resect stage 3 or 4 tumors that are localized. The patients with localized tumors included in this study were referred by urologists for systemic therapy, either because the patient was too sick or physically unable to undergo surgery, or because the kidney lesion was too difficult to remove and systemic therapy was administered with the intent to shrink the tumor prior to surgery. Patients were excluded if pregnant. Within four weeks before starting systemic therapy, patients underwent a CT perfusion and a staging CT scan of the chest, abdomen and pelvis (baseline). Each patient subsequently received systemic treatment with an angiogenesis inhibitor (bevacizumab, pazopanib, sunitinib). After eight days (range 6–13 days) of treatment, a second CT perfusion acquisition was obtained. After a standard of care interval of approximately 90 days of treatment, a re-staging CT scan of the chest, abdomen and pelvis was performed for RECIST measurements to determine response to therapy.

### 4.2. CT Perfusion Technique

Each patient underwent a CT perfusion acquisition of either the primary renal mass (if present) or the largest metastatic tumor (≥2.0 cm) in the chest, abdomen, or pelvis. Each CT scan was performed on a SOMATOM Force CT scanner (Siemens Healthineers, Erlangen, Germany). In each scan, a low-dose, non-contrast CT scan was obtained through the region of interest to localize the tumor. CT perfusion images were acquired at 80 kV and 100 mAs using an adaptive 4D spiral mode with variable pitch and *z*-axis coverage of 114–224 mm, depending on the size of the area of interest around the tumor). Mean CTDI_vol_ of 52.21 mGy (±18.8) and DLP of 992.68 mGy*cm (± 421.3) were utilized. Intravenous contrast (iohexol 370 or iohexol 300 if the patient had undergone a previous nephrectomy) was administered through an antecubital vein at 5 cc/sec for a total of 50 cc followed by a 45 cc saline chaser at 5 cc/s. After a scan delay of 7 s, images were obtained every 1.5 s for 20 acquisitions, followed by every 3.0 s for another five acquisitions, resulting in a total acquisition time of 45 s. All patients were instructed to hold their breath as long as possible after inhalation. If they were unable to hold their breath for the entire scan duration, patients were instructed to take slow shallow breaths in order to minimize motion. Each examination was monitored by a radiologist with >10 years of experience expertise in CT perfusion.

### 4.3. CT Perfusion Analysis

Each CT perfusion examination was post-processed and tumors were segmented on syngo.via VB10/VB20 (Siemens Healthineers, Erlangen, Germany) by two radiologists in consensus. Perfusion parameters were obtained using a deconvolution method following non-rigid body motion correction and 4D noise reduction. Arterial input function was obtained over the aorta or the largest artery near the tumor. Perfusion parameters included blood volume (BV), blood flow (BF), and flow extraction product (FEP). For perfusion analysis, 5 mm thick slices were used.

### 4.4. Treatment Response

The longest tumor dimension was measured in each lesion. Clinical response was defined as progressive disease, stable disease, partial response, or complete response based on RECIST 1.1 after 12 weeks of treatment. Progressive disease was defined as a ≥ 20% increase in the sum of target lesion diameters; stable disease was a < 30% decrease or a < 20% increase in the sum of target lesion diameters; partial response was defined as a ≥ 30% decrease in the sum of target lesion diameters; complete response was defined as disappearance of all lesions.

### 4.5. Analysis

We used descriptive statistics and graphical measures to assess if early changes in CT perfusion parameters (BF, BV, FEP) could be observed at day eight after the initiation of treatment. We used t-tests or Wilcoxon rank sum tests to compare the distribution of each CT perfusion parameter and tumor size by tumor location. Specifically, we compared the following measures by tumor location: Tumor size and individual perfusion parameters at baseline and at day eight as well as the difference in each measure from baseline to day eight. For patients with multiple lesions per organ, data from the largest lesion in the organ were used in our analysis. To evaluate the clinical response, we combined the treatment response categories into two groups: Stable or progressive disease and conducted univariable exact logistic regression to assess the association between clinical response and each individual measure for each tumor location separately (primary kidney mass or metastasis). Univariable exact logistic regression analysis was also used to determine the association of clinical response and tumor location. Significance testing was assessed at a two-sided alpha level of 0.10.

## 5. Conclusions

In conclusion, it is possible to perform one-week CT perfusion acquisitions in patients with RCC undergoing systemic treatment. We selected RCC as an optimal patient population to examine early changes in tumor perfusion due to the broad use of angiogenesis inhibitors for systemic therapy. There appear to be detectable changes in perfusion measurements in both primary and metastatic tumor sites as early as one week after initiating treatment. Future studies are planned to more fully evaluate the clinical potential for CT perfusion for early therapeutic monitoring.

## Figures and Tables

**Table 1:** Patient Demographics, tumor type and location.

Mean Age	53.5
Gender	4F/6M
Tumor Type	clear cell
Tumor Location	5 patients (pts) with primary renal masses 4 pts with RCC metastasis (single or multiple) 1 pt with primary RCC and 1 metastasis
Treatment Type	2 axitinib 2 sunitinib 5 pazopanib 1 sunitinib and gemcitabine

**Table 2:** CT perfusion parameter values by tumor location.

Parameter	Primary Renal Mass (*n* = 6)			Metastatic Mass (*n* = 7)		
	Baseline	Day 8	Difference	Baseline	Day 8	Difference
**Blood volume**						
Mean (SD)	13.4 (8.20)	8.1 (5.61)	−5.3 (3.25)	17.3 (9.4)	11.3 (8.60)	−6.1 (7.1)
Median (IQR)	15.9 (6.0, 17.9)	8.2 (3.0, 12.9)	−5.6 (−8.1, −3.0)	15.6 (10.1, 27.8)	7.7 (4.2, 18.2)	−3.2 (−11.9, −0.03)
**Blood flow**						
Mean (SD)	125.4 (70.5)	99.8 (73.0)	−25.5 (34.51)	235.7 (185.05)	160.7 (159.70)	−75.0 (80.47)
Median (IQR)	148.5 (64.0, 163.1)	97.8 (37.3, 140.0)	−15.7 (−26.7, −3.8)	148.4 (120.4, 352.3)	94.2 (48.7, 233.6)	−99.7 (−118.7, −1.0)
**Flow extraction product**						
Mean (SD)	60.6 (34.18)	39.9 (27.14)	−20.7 (17.91)	61.5 (28.70)	34.0 (24.43)	−27.5 (21.43)
Median (IQR)	74.8 (31.4, 79.1)	44.0 (13.0, 56.6)	−20.1 (−31.3, −2.09)	61.6 (34.5, 79.8)	28.6 (17.8, 64.7)	−40.1 (−42.5, 0.12)

Blood volume (mL/100 mL), blood flow (mL/100 mL/min), flow extraction product (mL/100 mL/min); SD = standard deviation; IQR = interquartile range.
